# Supplementary material for: Immunomodulatory Effect of Isocaloric Diets with Different Protein Contents on Young Adult Sprague Dawley Rats
Source: Foods. 2023 Apr 10;12(8):1597. doi: 10.3390/foods12081597 (PMC10138247; doi:10.3390/foods12081597)
Supplement: Supplementary file 1 [file foods-12-01597-s001.zip › foods-2312381-supplementary.pdf]

Table S1. The amino acid content of diets differing in CP contents

Amino acid[1]	Diets					
	10% CP	14% CP	20% CP	28% CP	38% CP	50% CP
	Amino acid content (g/100g)					
Lys	1.28	1.14	1.19	1.59	2.11	3.17
Met	0.70	0.60	0.65	0.87	1.11	1.68
Thr	0.76	0.71	0.79	1.03	1.30	1.88
Phe	0.50	0.61	0.93	1.33	1.68	2.41
Val	0.46	0.59	0.76	1.36	1.73	2.56
Ile	0.39	0.50	0.83	1.17	1.49	2.22
Leu	0.97	1.19	1.87	2.51	3.14	4.49
Asp	0.75	0.87	1.29	1.81	2.19	3.13
Ser	0.46	0.56	0.89	1.25	1.57	2.27
Glu	1.75	2.19	3.44	5.05	6.44	9.34
Gly	0.38	0.39	0.50	0.62	0.69	0.92
Ala	0.54	0.59	0.81	1.00	1.17	1.57
Tyr	0.34	0.45	0.72	1.10	1.42	2.13
His	0.27	0.33	0.50	0.72	0.91	1.30
Arg	1.05	0.97	1.02	1.04	1.23	1.68
Pro	0.85	1.12	1.96	3.10	4.21	6.09
Cys	0.12	0.102	0.120	0.14	0.14	0.18

[1] Determined values. Animo acid contents were measured using the Hitachi L-8800 amino acid analyzer (Tokyo, Japan).

Abbreviation: CP, Crude protein.

Table S2. Effects of feeding diets with different protein contents for 6 weeks on growth performance

Items	Treatments						SEM	*P*-value
	10% CP	14% CP	Control	28% CP	38% CP	50% CP		
initial body weight (g)	556	551	521	550	528	570	31.0	0.881
final body weight (g)	1343	1433	1530	1350	1412	1357	62.7	0.290
total food intake (g)	4607	4696	4739	4515	4329	4312	175	0.393
food intake (g/day)	110	112	113	108	103	103	4.17	0.392
body weight gain (g/day)	18.8^{b}	21.1^{ab}	24.0^{a}	19.0^{b}	21.0^{ab}	18.7^{b}	1.16	0.020
protein intake (g/day)	13.1^{e}	15.7^{e}	23.0^{d}	29.9^{c}	39.3^{b}	49.8^{a}	1.29	< 0.001
body weight gain (g)/g protein intake	1.44^{a}	1.35^{a}	1.05^{b}	0.63^{c}	0.54^{c}	0.38^{d}	0.043	< 0.001

^{a,b,c,d,e} Values within a row with different superscripts differ significantly. ($P < 0.05$)

Abbreviations: CP, Crude protein; SEM, Standard error of measurement.

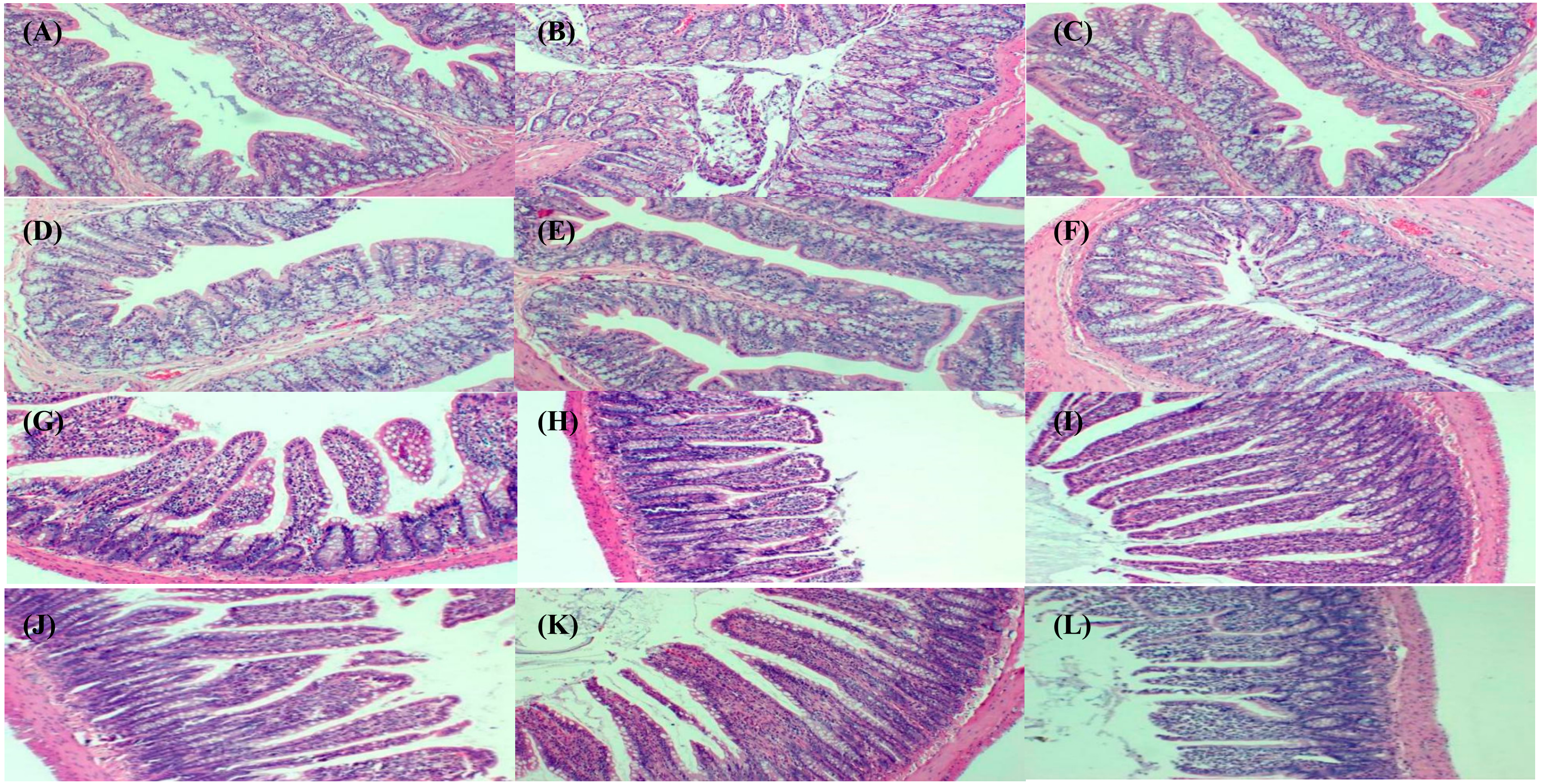
(A)
(B)
(C)
(D)
(E)
(F)
(G)
(H)
(I)
(J)
(K)
(L)

Figure S1. Effects of isocaloric diets with high or low protein for 6 weeks on colon (**A**, **B**, **C**, **D**, **E**, **F**) and ileum (**G**, **H**, **I**, **J**, **K**, **L**) morphology by magnification 200 × in the experimental rats. (**A**, **G**) the 10% CP group; (**B**, **H**) the 14% CP group; (**C**, **I**) the 20% CP group; (**D**, **J**) the 28% CP group; (**E**, **K**) the 38% CP group; and (**F**, **L**) the 50% CP group.
